# An Assessment of Early Response to Targeted Therapy via Molecular Imaging: A Pilot Study of 3′-deoxy-3′[(18)F]-Fluorothymidine Positron Emission Tomography ^18^F-FLT PET/CT in Prostate Adenocarcinoma

**DOI:** 10.3390/diagnostics7020020

**Published:** 2017-04-04

**Authors:** Kalevi Kairemo, Gregory C. Ravizzini, Homer A. Macapinlac, Vivek Subbiah

**Affiliations:** Department of Nuclear Medicine, The University of Texas MD Anderson Cancer Center, 1400 Pressler Street, Unit 1483, FCT 16.6005, Houston, TX 77030, USA; GRavizzini@mdanderson.org (G.C.R.); hmacapinlac@mdanderson.org (H.A.M.); vsubbiah@mdanderson.org (V.S.)

**Keywords:** fluorine-18 fluorothymidine (^18^F-FLT), prostate cancer, positron emission tomography/computerized tomography (PET/CT), molecular imaging

## Abstract

Fluorothymidine is a thymidine analog labeled with fluorine-18 fluorothymidine for positron emission tomography (^18^F-FLT-PET) imaging. Thymidine is a nucleic acid that is used to build DNA. Fluorine-18 fluorothymidine (^18^F-FLT) utilizes the same metabolic pathway as does thymidine but has a very low incidence of being incorporated into the DNA (<1%). ^18^F-FLT-PET could have a role in the evaluation of response to targeted therapy. We present here a pilot study where we investigated cellular metabolism and proliferation in patients with prostate cancer before and after targeted therapy. Seven patients with Stage IV prostate adenocarcinoma, candidates for targeted therapy inhibiting the hepatocyte growth factor/tyrosine-protein kinase Met (HGF/C-MET) pathway, were included in this study. The HGF/C-MET pathway is implicated in prostate cancer progression, and an evaluation of the inhibition of this pathway could be valuable. ^18^F-FLT was performed at baseline and within four weeks post-therapy. Tumor response was assessed semi-quantitatively and using visual response criteria. The range of SUVmax for ^18^F-FLT at baseline in the prostate varied from 2.5 to 4.2. This study demonstrated that ^18^F-FLT with positron emission tomography/computerized tomography (^18^F-FLT PET/CT) had only limited applications in the early response evaluation of prostate cancer. ^18^F-FLT PET/CT may have some utility in the assessment of response in lymph node disease. However, ^18^F-FLT PET/CT was not found to be useful in the evaluation of the prostate bed, metastatic skeletal disease, and liver disease.

## 1. Introduction

Prostate cancer is the second most common cancer in men worldwide, affecting approximately one in every seven men [1]. Prostate cancer is diagnosed mostly in older men and is uncommon before age 40. In the United States, there will be an estimated 181,000 new cases and 26,100 deaths in 2016 [2]. Despite a wide range of imaging techniques such as ultrasound, computerized tomography (CT), magnetic resonance imaging (MRI), and positron emission tomography (PET), assessing prostate cancer remains a clinical challenge in many important phases of the disease. The most commonly utilized PET radiotracer in oncology, fluorine-18 fluorodeoxyglucose (^18^F-FDG), is not useful in diagnosis and the initial staging of clinically organ-confined disease [3,4,5]. Moreover, ^18^F-FDG may have a limited role in the assessment of occult disease at the time of biochemical recurrence [6] or in the evaluation of response to therapy of known metastatic disease, except in a few cases [7].

In this pilot study, we evaluated fluorine-18 fluorothymidine with positron emission tomography/computerized tomography (^18^F-FLT PET/CT) for imaging of prostate cancer in patients undergoing treatment as a potential imaging biomarker for assessing response to therapy. Fluorine-18 fluorothymidine (^18^F-FLT) is a structural analog of the DNA component, thymidine; however, it is not incorporated into the DNA. It is entrapped in the cell due to phosphorylation by thymidine kinase, a part of the proliferation pathway. In this sense, it is similar to ^18^F-FDG, since the tracer accumulates in the cell via the same mechanism as the physiological analog, but cannot be further metabolized and is hence “trapped” and continues to accumulate intracellularly. ^18^F-FLT is a marker of tumor proliferation and its uptake has been shown to be proportional to the DNA synthesis rate and proliferative indices [8]. Therefore, imaging of cellular proliferation could become an important diagnostic tool to evaluate the tumor growth rates and objectively assess potential response to treatment [9]. So far, only preclinical experiments evaluated the ^18^F-FLT effectiveness in prostate cancer animal models, but they have not been assessed in early therapy response. Pharmacokinetics of ^18^F-FLT and ^18^F-FDG, ^11^C-choline was compared in two hormone-independent (PC-3 and DU145) and two hormone-dependent (CWR22 and PAC 120) prostate cancer xenograft mouse models using PET [10]. The highest uptake of both ^18^F-FLT and ^18^F-FDG was in PC-3 tumors. Interestingly, uptake of ^18^F-FLT was insufficient to provide reliable information on response to therapy in the CWR22 tumor model. [11]. Another study reported a significant decline in ^18^F-FLT micro-PET uptake in the 22Rv1 hormone-refractory prostate tumor model, where tumors were implanted in athymic mice after treatment with docetaxel [12]. Therefore, the usefulness of ^18^F-FLT in the evaluation of treatment response in patients with prostate cancer is largely unknown, even though many PET tracers have been characterized for the clinical evaluation of prostate cancer [12]. This is to our knowledge the first report of using ^18^F-FLT in the assessment of therapy response in prostate cancer.

## 2. Methods

This prospectively designed, single-institution study was approved by the local institutional review board and was compliant with the Health Insurance Portability and Accountability Act. Written informed consent was obtained from each participant. Seven participants with histologically proven prostate adenocarcinoma were enrolled. A baseline ^18^F-FLT PET/CT was obtained before treatment with MET inhibitor followed by a second ^18^F-FLT PET/CT approximately 28–29 days after treatment in all participants. The mean age of the participants was 65 years (range, 51–74 years) and serum prostate specific antigen (PSA) concentration ranged from 0.2 to 820 ng/mL.

### 2.1. Radiosynthesis of ^18^F-FLT

The radiosynthesis of ^18^F-FLT was produced according to a method already described [13,14,15]. Briefly, fluorine-18 fluoride is prepared by the ^18^O (p, n) ^18^F reaction using >95% ^18^O enriched water as the target material with a titanium window and target holder. The enriched water is bombarded with 17 MeV protons at 30 microamps. At the end of bombardment, the target water is pushed out of the target by helium pressure into the hot cell where the fluorine-18 fluoride is trapped on an anion exchange resin. The ^18^F fluoride is then eluted with [2.2.2.] Kryptofix^®^ (Sigma-Aldrich, Gillingham, UK)/potassium carbonate solution and the fluoride is dried by azeotropic distillation with acetonitrile. The ^18^F fluoride is then reacted with the 5′-benzoate of 2,3′-anhydrothymine in DMSO at 160 °C [15]; then, after cooling down to 50 °C, 1% NaOH (0.35 mL) heated to 50 °C, 0.2 M NaH_2_PO_4_ (0.75 mL), and 1.5 mL of 8% EtOH/92% 0.01 M NaH_2_PO_4_ are added before passing through an alumina cartridge and loaded on a preparative HPLC column. The FLT is purified by high-performance liquid chromatography on an ^18^C reversed phase column using 8% ethanol/92% 0.01 M NaH_2_PO_4_ as the mobile phase. The yield is typically 20–50 mCi, and the specific activity is 1–4 Ci/mmol.

### 2.2. Patient Selection, Preparation, and Clinical Trial Description

All patients had a diagnosis of Stage IV prostate cancer and were refractory to standard lines of therapy. We reviewed the medical records of patients with advanced cancer who had functional imaging as part of their care at MD Anderson. This study was performed in accordance with the guidelines of the MD Anderson Institutional Review Board (IRB). Because this was a retrospective chart review IRB has waived the consent requirements. They were enrolled on c-MET based trials available in the institution. After a washout period from progression of standard care therapy, patients were enrolled on the clinical trial targeting the HGF/MET pathway. Patients had baseline scans that included CT of the chest, abdomen, and pelvis and a bone scan. This was primarily used for an evaluation of response. FLT studies were performed before the initiation of the study drug, and images were obtained at least 28 days after therapy. Patients fasted for 3 h prior to ^18^F-FLT administration. 

### 2.3. The Positron Emission Tomography/Computerized Tomography (PET/CT) Study

PET/CT studies were performed using a Discovery ST8 PET/CT system (GE Healthcare, Milwaukee, WI, USA) in combination with the CT component of an 8-MDCT scanner (LightSpeed, GE Healthcare). A single-dose injection of 184–360 MBq (4.96–9.72 mCi) of ^18^F-FLT was administered intravenously. Whole-body PET imaging (WB PET) consisted of 4 or 5 bed positions, 10 min per bed position) approximately 60 min after radiotracer injection. PET images were reconstructed using standard vendor-provided reconstruction algorithms. The CT component of the study consisted of a helical scan covering the head to the mid-thighs (120 kVp, 300 mA, 0.5-s rotation; table speed, 13.5 mm/rotation) with no contrast enhancement. Axial CT images were reconstructed with a slice thickness of 3.75 mm. The PET projection data were corrected for random coincidences, scatter, and attenuation. Transaxial images were reconstructed into 128 × 128 pixel images with a pixel size of at least 4.5 mm. PET images were reconstructed using standard vendor-provided reconstruction algorithms that incorporate ordered-subset expectation maximization and were corrected for attenuation using data from the CT component of the examination; emission data are corrected for scatter, random events, and dead-time losses as well using the PET/CT scanner’s standard algorithms. The dose calibrators (CRC-15R; Capintec, Florham Park, NJ, USA) were cross-calibrated with the PET/CT measuring instrument to ensure quantitative accuracy of the PET data. Measurements of uptake and retention in tumors were obtained from the WB PET acquisition and compared to normal tissue.

### 2.4. Image Analysis

Regional whole-body reconstructed PET/CT data are stored in the Digital Imaging and Communications in Medicine 3.0, part 10, file format and transferred to a PET/CT image analysis workstation. Three-dimensional volumes of interest (VOIs) of identifiable primary tumors and metastases and source organs will be constructed on the CT images, and their positions verified on the corresponding PET images to include all organ activity. These VOIs were then be used for PET image analysis. The identifiable source organs analyzed were the heart, liver, gallbladder, kidneys, urinary bladder, small and large intestines, brain, and whole body. Three-dimensional VOI definitions will be used to visually inspect for mis-registration due to motion between sequential scans of the same segment. Residual errors were manually corrected by redefining the VOIs when necessary; this is necessary only for the gall bladder and urinary bladder, in the event that gradual accumulation of radioactivity as well as enlargement over the course of the PET scan occurs.

### 2.5. ^18^F-FLT PET/CT Scan Interpretation

Blinded image interpretation was performed by two experienced nuclear medicine specialists (>15 years of experience each, Kalevi Kairemo, Gregory C. Ravizzini). ^18^F-FLT PET/CT uptake of target lesions was evaluated visually as present or absent. Certainty of the findings was graded on a 3-point scale. Only after the scans were visually interpreted and the final interpretation/score entered for each patient did these specialists determine the maximum SUV for each residual mass/lesion by CT, regardless of whether it was FLT-PET-negative. In addition, the mean SUV of the normal liver was determined.

## 3. Results

**Figure 1:**
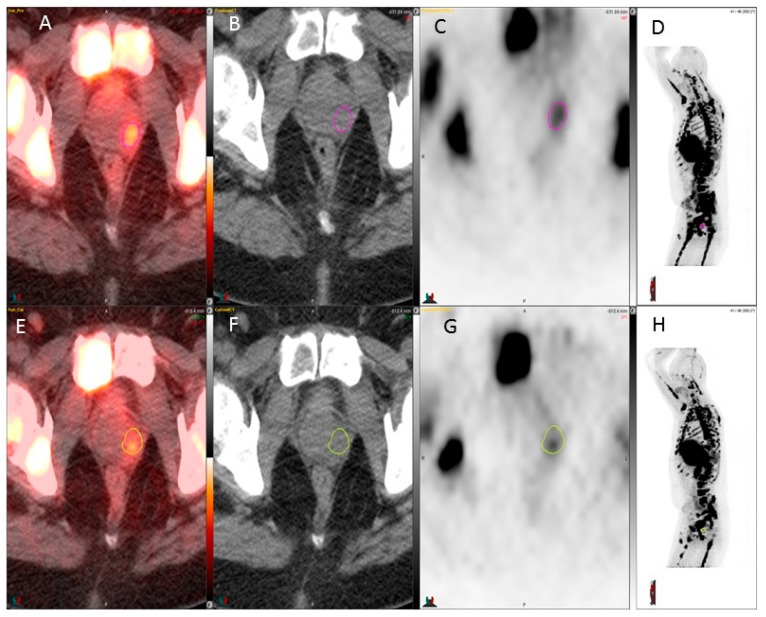
^18^F-FLT used for detection of prostate foci before and after chemotherapy. A weak response is seen, while the SUVmax value decreased from 3.6 to 2.3 in the left prostate lobe (circles in **C** and **G**). Upper row, baseline: from the left fusion image (**A**), computerized tomography (CT) image (**B**), fluorine-18 fluorothymidine with positron emission tomography (^18^F-FLT PET) image (**C**); and maximum intensity projection (MIP) image (**D**); lower row, after 4 weeks of therapy: from the left fusion image (**E**), CT image (**F**), ^18^F-FLT PET image (**G**), and MIP image (**H**). Color bar range from 0 to 5.

**Figure 2:**
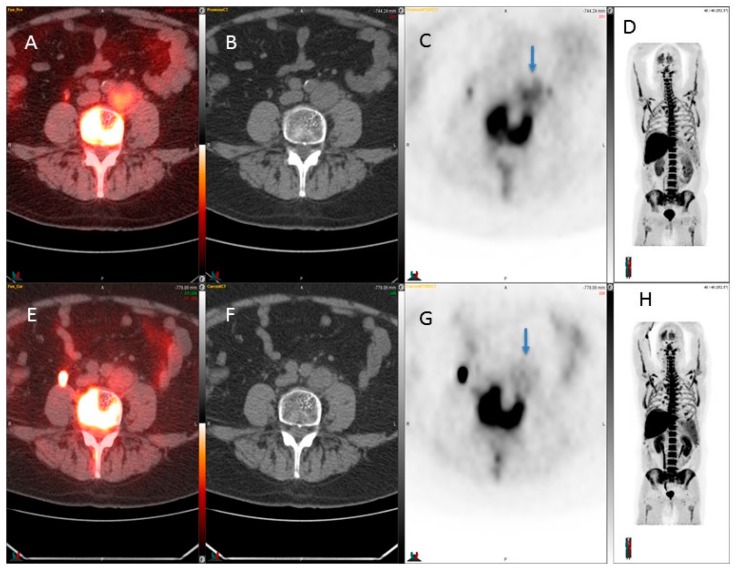
^18^F-FLT used for detection of retroperitoneal lymph node metastases before and after chemotherapy. A weak response was seen while the SUVmax value decreased from 4.1 to 2.1 in the left common iliac node (arrows in **C** and **G**). Upper row, baseline: from the left fusion image (**A**), CT image (**B**), ^18^F-FLT PET image (**C**), and MIP image (**D**); lower row, after 4 week therapy: from the left fusion image (**E**), CT image (**F**), ^18^F-FLT PET image (**G**), and MIP image (**H**). Color bar range from 0 to 5.

**Figure 3:**
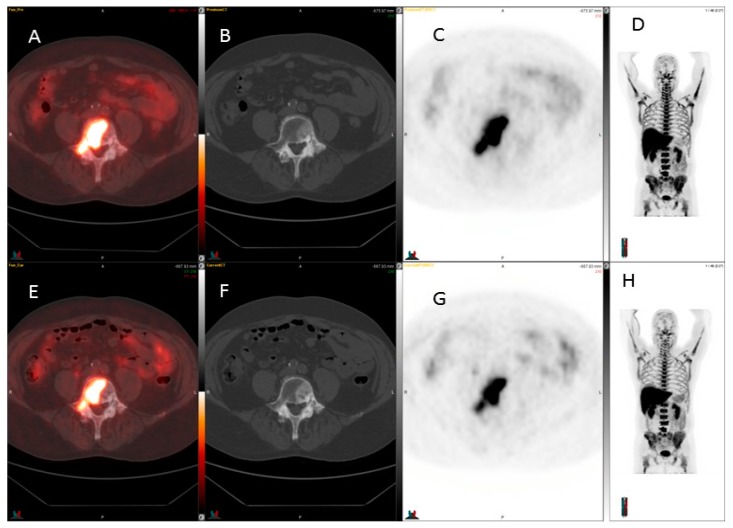
^18^F-FLT detection of prostate cancer metastases before and after therapy. The sclerotic skeletal metastases seen on CT are seen as defects on ^18^F-FLT, so the usefulness of ^18^F-FLT for detection of skeletal metastases is still unclear since visualization of lesions may be hindered by prominent background activity in the bone marrow. The skeletal metastases visualize most often as defects. Upper row, baseline: from the left fusion image (**A**), CT image (**B**), ^18^F-FLT PET image (**C**), and MIP image (**D**); lower row, after 4 week therapy: from the left fusion image (**E**), CT image (**F**), ^18^F-FLT PET image (**G**), and MIP image (**H**). Color bar range from 0 to 5.

The FLT PET kinetics are tabulated in Table 1. Figure 1 and Figure 2 show ^18^F-FLT images and show weak positivity. Figure 3 shows skeletal metastases as defects. In the seven patients, the SUVmax of the ^18^F-FLT in the prostate varied from 2.5 to 4.2 at baseline. One patient (#1) had lesions in both prostate lobes, and the lesion found on the left decreased in activity (Figure 1), whereas the lesion on the right increased in activity. This patient had PSA progression (Table 1).

A weak response was seen in Patient #3 while the SUVmax value decreased from 4.1 to 2.1 in the left common iliac node, i.e., −49 % (Figure 2). This common iliac node also decreased in size, from 3.6 cm down to 3.1 cm, i.e., −14%. This patient also had PSA response (Table 1). ^18^F-FLT was used for detection of retroperitoneal lymph node metastases before and after chemotherapy, and two other patients did not show any significant (>30%) ^18^F-FLT change.

Three patients demonstrated skeletal disease, mainly as defects. An example is shown in Figure 3, who had an active skeletal disease. It was visualized on also CT. In one patient, a decrease in bone marrow could be observed, even though this patient had a disease progression. Cortical bone lesions could not be assessed by ^18^F-FLT-PET. Two patients had known liver metastases: in the patient with a single lesion, the activity increased, whereas in the patient with multiple lesions, the metastases were visualized as defects and could not be assessed for activity.

## 4. Discussion

Due to the known limitations of ^18^F-FDG in the evaluation of patients with prostate cancer, the development of new imaging biomarkers targeting this disease has been a subject of intense study by many groups. Recently, ^11^C choline and ^18^F-FACBC have been approved by the US Food and Drug Administration for PET imaging in patients with suspected prostate cancer recurrence (biochemical recurrence) and non-informative bone scintigraphy, CT, or MRI. The biological basis for the accumulation of radio-labeled choline in tumors is a reflection of the overexpression of choline kinase in support of malignancy-induced increased demand for cellular membrane synthesis [16]. As an example of ^11^C-choline, sensitivity, and specificity for the detection of primary prostate cancer is 87% and 62%, respectively, in reference to histopathologic examination [17]. 

Another approach of specific localization for prostate cancer is the use of radiolabeled monoclonal antibodies and peptides targeted against specific cell surface antigens, namely, the prostate-specific membrane antigen (PSMA) [18]. PSMA is a 100 kDa type 2 transmembrane glycoprotein expressed in prostate epithelial cells. The biomarker is 94% extracellular and contains short internal and transmembrane domains [19]. Expression is minimal in the normal prostate gland, but amplified in both localized and metastatic prostate cancer. PSMA expression correlates directly with tumor grade and is also significantly upregulated in castrate-resistant prostate cancer. Radiotracer ^111^In-capromab pendetide recognizes PSMA epitope in the intracellular domain of PSMA and is clinically approved by the FDA for pre-surgical staging or evaluation of biochemical recurrence after local therapy [20]. A radiolabeled peptide labeled with Gallium-68, Glu-NH-CO-NH-Lys-(Ahx)-[(HBED-CC)], binds to the site on the exterior of the cell and is routinely used in Europe for PET/CT imaging. Targeting of external epitopes of PSMA enhances availability of the antigen and tumor detectability [21]. The first report evaluating Glu-NH-CO-NH-Lys-(Ahx)-[^68^Ga(HBED-CC)] (^68^Ga-PSMA) came from Afshar-Oromieh et al. [22]. This novel PET/CT peptide-based radiotracer was able to detect prostate cancer relapse and metastases with significantly high uptake.

The FLT marker is less affected by post-therapy inflammatory changes due to macrophages/monocyte infiltration that is seen as increased FDG uptake. This may be explained by the fact that immigrant macrophages/monocytes do not proliferate, and since FLT measures cell proliferation, no uptake (or only negligible uptake) of this tracer is expected in the inflammatory lesions. One study compared both tracers in Wistar rats bearing C6 rat glioma in the shoulder with a sterile inflammatory lesion in the calf muscle induced by turpentine [23]. Both FDG and FLT accumulated in the tumor. However, only FDG accumulated in the inflamed muscle, whereas FLT did not, confirming the higher tumor specificity of FLT in this animal model. This higher tumor-specificity is very likely to prove advantageous in response assessment following treatment, especially early on when the inflammatory changes have not yet subsided, or following RT or combined modality therapy, where post-therapy inflammatory changes may be observed for 2–3 months or longer. 

There is currently limited data in regard to the utility of 3′-deoxy-3’-^18^F-fluorothymidine (^18^F-FLT) in the management of prostate cancer. ^18^F-FLT is a structural analog of the DNA constituent, thymidine. ^18^F-FLT is not incorporated into DNA, but is trapped in the cell due to phosphorylation by thymidine kinase, a part of the proliferation pathway. In this sense, it is similar to ^18^F-FDG: the tracer accumulates in the cell via the same mechanism as the physiological analog, but cannot be further metabolized and is hence “trapped” and continues to accumulate intracellularly. Analogous to ^18^F-FDG which is a marker of glucose utilization, ^18^F-FLT is a marker of tumor proliferation and its uptake has been shown to be proportional to the DNA synthesis rate and proliferative indices [10].

Oyama et al. [12] evaluated the ability of ^18^F-FLT to assess early therapeutic effects of androgen deprivation in an animal tumor model of prostate cancer. In this study, subcutaneous tumor xenografts were created in athymic mice using the human androgen-dependent cell line CWR22. Biodistribution studies demonstrated that ^18^F-FLT is rapidly taken up by the tumor and is retained longer than in other tissues. In addition, there was a significant decrease in ^18^F-FLT uptake in mice treated with the androgen deprivation drug diethylstilbestrol in comparison with control mice. Even though the authors concluded that ^18^F-FLT may be useful in the evaluation of changes in the proliferation activity of prostate cancer during AD therapy, many questions remain. For example, the usefulness of ^18^F-FLT for detection of skeletal metastases is still unclear since visualization of lesions may be hindered by prominent background activity in the bone marrow [24,25,26].

Limitations exist in this small pilot trial. First, this was a retrospective review of patients that received a targeted therapy. Since responses were rarely seen and this is a negative study, it is quite challenging to publish negative data. Secondly, this pilot study selected only prostate cancer patients. We do not know the expression of c-MET in these patients. What is known from a literature review is that c-Met’s overexpression is noted in both primary prostate cancers and bone metastases [27,28,29]. Moreover, its expression has been correlated with castrate-resistant disease. Because of this, several inhibitors are in different stages of clinical development [28,30,31,32].

This is the first clinical report of using ^18^F-FLT in prostate cancer. Numerous other reports exist in other diseases where this method works. Unfortunately, in prostate cancer, ^18^F-FLT PET seems to have limited value. Of two patients with prostate lesions, the first demonstrated a mixed response (decrease/increase) and the second a slight decrease in prostate activity, but the disease progressed in both patients. Three patients had lymph node disease. Only one patient responded, based on PSA change, and this patient also demonstrated a significant decrease (−51%) in lymph node activity, whereas two patients whose disease progressed did not demonstrate any significant changes in the SUVs of ^18^F-FLT (Table 1). Three patients had advanced skeletal disease, and it was visualized as defects on ^18^F-FLT PET scans, probably due to prostate cancer cell invasion in the bone marrow. Similarly, multiple liver metastases were visualized as defects. ^18^F-FLT PET may play a role in oligometastatic conditions both in the skeleton and in the liver, but we only had one example and no conclusions can be drawn (Patient #1, Table 1). Future studies are warranted to compare ^11^C choline, ^18^F-FACBC, and ^18^F-FLT PET together with conventional scans such as bone scintigraphy, CT, and MRI.

## 5. Conclusions

This pilot study of ^18^F-FLT PET/CT shows that it has only limited applications in the early response evaluation of prostate cancer. Although it may be useful in the assessment of response in lymph nodes (Figure 2), it may not be effective in the evaluation of the prostate bed (Figure 1), advanced skeletal disease, and liver disease.

## Figures and Tables

**Table 1:** Positron emission tomography/computerized tomography (PET/CT) changes in patients with prostate cancer on MET inhibitor therapy. Fluorine-18 fluorothymidine (^18^F-FLT) SUVmax value changes are presented at baseline and after 4 weeks of treatment in different organs. Ø: No findings.

Patient/Age	Prostate SUVmax BL-> 4 Weeks	Lymph Nodes SUVmax BL-> 4 Weeks Size (cm) BL-> 4 Weeks	Skeletal Disease SUVmax BL-> 4 Weeks	Soft Tissue	PSA Response
1/67	2.5→2.8 3.6→2.3	1.6→1.5 1.1 × 1.0→1.0 × 0.9 (para-aortic)	1.6→1.5 (cortex) 6.8→5.2 (marrow)	9.8→12.8 (liver)	0.2→0.5
2/74	Ø	Ø	defects	Ø	387→871
3/65	Ø	4.1→2.4 3.6 × 3.4→3.1 × 2.6 (iliaca comm L)	Ø	Ø	95→62
4/51	4.2→3.9	4.3→3.6 3.7 × 4.4→3.3 × 4.2 (iliaca comm R)	Ø	Ø	820→2138
5/72	Ø	Ø	defects	Ø	47.8→98.0
6/74	Ø	Ø	defects	Ø	3.1→6.3
7/61	Ø	Ø	Ø	liver defects	16.6→18.8
